# *Drosophila* Vps13 Is Required for Protein Homeostasis in the Brain

**DOI:** 10.1371/journal.pone.0170106

**Published:** 2017-01-20

**Authors:** Jan J. Vonk, Wondwossen M. Yeshaw, Francesco Pinto, Anita I. E. Faber, Liza L. Lahaye, Bart Kanon, Marianne van der Zwaag, Antonio Velayos-Baeza, Raimundo Freire, Sven C. van IJzendoorn, Nicola A. Grzeschik, Ody C. M. Sibon

**Affiliations:** 1 Department of Cell Biology, University Medical Center Groningen, University of Groningen, Groningen, the Netherlands; 2 Wellcome Trust Centre for Human Genetics, Oxford, United Kingdom; 3 Unidad de Investigación, Hospital Universitario de Canarias, Instituto de Tecnologías Biomédicas, Ofra s/n, La Laguna, Tenerife, Spain; CINVESTAV-IPN, MEXICO

## Abstract

Chorea-Acanthocytosis is a rare, neurodegenerative disorder characterized by progressive loss of locomotor and cognitive function. It is caused by loss of function mutations in the *Vacuolar Protein Sorting 13A* (*VPS13A*) gene, which is conserved from yeast to human. The consequences of VPS13A dysfunction in the nervous system are still largely unspecified. In order to study the consequences of VPS13A protein dysfunction in the ageing central nervous system we characterized a *Drosophila melanogaster Vps13* mutant line. The *Drosophila Vps13* gene encoded a protein of similar size as human VPS13A. Our data suggest that Vps13 is a peripheral membrane protein located to endosomal membranes and enriched in the fly head. *Vps13* mutant flies showed a shortened life span and age associated neurodegeneration. *Vps13* mutant flies were sensitive to proteotoxic stress and accumulated ubiquitylated proteins. Levels of Ref(2)P, the *Drosophila* orthologue of p62, were increased and protein aggregates accumulated in the central nervous system. Overexpression of the human Vps13A protein in the mutant flies partly rescued apparent phenotypes. This suggests a functional conservation of human VPS13A and *Drosophila* Vps13. Our results demonstrate that Vps13 is essential to maintain protein homeostasis in the larval and adult *Drosophila* brain. *Drosophila Vps13* mutants are suitable to investigate the function of Vps13 in the brain, to identify genetic enhancers and suppressors and to screen for potential therapeutic targets for Chorea-Acanthocytosis.

## Introduction

Chorea-Acanthocytosis (ChAc, MIM 200150) is a rare neurodegenerative disorder characterized by chorea, orofacial dyskinesia and psychiatric symptoms including tics (reviewed in [1,2]). In addition to the neurological symptoms, spiky red blood cells (acanthocytes) are often observed. ChAc is a recessively inherited disease caused by mutations in the *VPS13A* gene, hereafter called *HsVPS13A* [3,4]. These mutations mostly lead to absence or reduced levels of the HsVPS13A (or also called chorein) protein [5]. Symptoms manifest on average at the age of 32 [1]. The pathophysiology of ChAc is largely unknown and it is not clear why HsVPS13A loss of function leads to the symptoms presenting in ChAc patients. *HsVPS13A* is evolutionarily conserved and orthologues are present in various organisms such as *Mus musculus*, *Drosophila melanogaster*, *Caenorhabditis elegans*, *Tetrahymena thermophila*, *Dyctiostelium discoidenum* and *Saccharomyces cerevisiae* [6–8].

HsVPS13A belongs to the VPS13 family of proteins, which in humans consists of four members, VPS13A to D. All members have an N-terminal chorein domain of unknown function. Besides HsVPS13A other members of this family are also associated with medical conditions. *VPS13B* mutations cause Cohen syndrome, a developmental disorder characterized by mental retardation, microcephaly and facial dysmorphisms [9]. VPS13B has been reported to be a Rab6 effector that controls Golgi integrity [10,11]. *VPS13C* mutations have recently been described to cause autosomal-recessive early-onset Parkinson’s disease, probably by alteration of mitochondrial morphology and function [12]. The VPS13C protein has also been suggested to play a role in adipogenesis [13]. Additionally, a number of genetic studies have found an association of *VPS13C* with glucose and insulin metabolism [14,15], and of *VPS13D* with altered interleukin 6 production [16].

Knowledge about the cellular function of the Vps13 protein family members mainly comes from investigations in *S*. *cerevisiae* were a single *VPS13* gene encodes a peripheral membrane protein [17], Vps13, which is involved in the trafficking of multiple proteins from the trans-Golgi network to the pre-vacuolar compartment [17,18]. Vps13 is also required for the formation of the prospore membrane by controlling the levels of phosphatidylinositol-4-phosphate [19]. Recently, it has been demonstrated that Vps13 is important for mitochondrial integrity and at least some functions of Vps13 are redundant with functions of ERMES, a protein complex that tethers the endoplasmic reticulum and the mitochondria [20,21]. Although ERMES plays an important role in yeast, so far no counterpart has been identified in metazoans.

In various organisms Vps13 function has been linked to lysosomal degradation pathways. In the ciliate *Tetrahymena thermophila* TtVPS13A is required for phagocytosis [7,22] and in *Dictyostelium discoideum* TipC, the HsVPS13A *Dictyostelium* orthologue, plays a role in autophagic degradation [8]. A role for HsVPS13A in autophagy has also been supported by experiments performed in human epitheloid cervix carcinoma cells, where knock down of HsVps13A leads to an impairment of the autophagic flux [8].

Studies to understand a possible function of VPS13A in the brain are limited. *Vps13A* knockout mice show recapitulation of some of the patient’s characteristics such as acanthocytic red blood cells and an altered gait at an older age. Additionally, gliosis and TUNEL positive cells are present in the brain of these mice [23]. However, it is reported that the severity and penetrance of neurological phenotypes in mouse models of ChAc are variable or absent depending on the genetic background of the strains [24]. Therefore, additional animal models are required to identify genetic modifiers and to further understand the role of VPS13A in an ageing brain.

To further study the cellular function of VPS13A in an aging, multicellular model organism with a complex central nervous system we used *Drosophila melanogaster*. We established a *Drosophila* model for ChAc which showed a reduced life span, decreased climbing ability and age-associated neurodegeneration. Additionally it showed sensitivity to proteotoxic stress and impaired protein homeostasis. The phenotypes of *Vps13* mutant flies were rescued by overexpression of the Human VPS13A protein, indicating a functional conservation of *Drosophila* Vps13 and HsVPS13A. Drosophila *Vps13* mutants will be valuable for further detailed studies to investigate the role of VPS13A in brain tissue and to screen for possible therapeutic strategies.

## Results

### Characterization of *Drosophila Vps13* mutant flies

**Fig 1:**
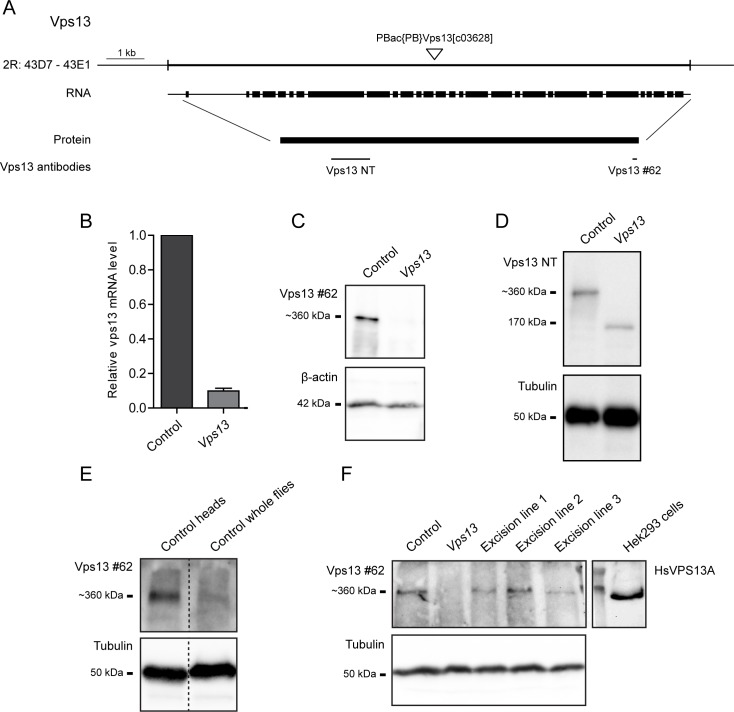
*Vps13*^*c03628*^ encodes for a truncated Vps13 protein. (A) Schematic representation of the *Vps13* gene and the genomic localization, RNA and protein is depicted. The epitopes of the polyclonal Vps13 antibodies (Vps13 NT and Vps13 #62) are indicated.(B) Relative levels of *Vps13* mRNA in control and *Vps13* mutant flies were determined by Q-PCR. Mean and SEM (n = 2) are plotted. (C) Western blot analysis of Vps13 protein in control and *Vps13* mutant fly heads using the Vps13 #62 antibody. β-Actin was used as a loading control. (D) Western blot analysis of the level of Vps13 protein in control and *Vps13* mutant fly head extracts analyzed with the Vps13 NT antibody. α-tubulin was used as a loading control. (E) Lysates of the heads of control flies and whole control flies were analyzed for Vps13 levels. α-tubulin was used as a loading control. (F) Lysates of the heads of control flies, *Vps13* mutant flies and three excision lines were analyzed for Vps13 levels. Human VPS13A was detected in samples of Hek293 cells. *Drosophila* samples and human samples were run on the same gel, separated by a lane containing the molecular weight standards, after transfer of the membrane, the marker lane was split to detect human and Drosophila VPS13 separately using species specific antibodies. The marker lane was used to align the blots after antibody detection. α-tubulin was used as a loading control.

ChAc is caused by mutations in the *VPS13A* gene [3,4], which lead to absence or reduced levels of HsVPS13A protein [5]. The *Drosophila* genome encodes for three predicted Vps13 proteins, orthologues to human VPS13A, B and D; in this study we focused on the structural orthologue of HsVPS13A, further referred to as Vps13 [6] (S1 Fig). The Exelixis *Drosophila* fly line *Vps13*^*c03628*^ carries a PiggyBac transposable element in an intronic region of the *Vps13* gene (Fig 1) [25]. Flies heterozygous for this mutation (*Vps13*^*-/+*^) did not show any mutant phenotype; homozygous mutants (*Vps13*^*-/-*^) were viable and were investigated further. Analysis by qPCR showed lower levels of *Vps13* mRNA in homozygous *Vps13* mutant flies (Fig 1). Polyclonal antibodies were raised against two different epitopes of the Vps13 protein (Fig 1). Both antibodies recognized a band in extracts from control fly heads (Fig 1), which migrated with the same mobility as the human protein in samples derived from HEK293 cells and detected with a HsVPS13A-specific antibody (Fig 1). Vps13 was highly enriched in samples from fly heads compared to samples from whole flies (Fig 1), suggesting that Vps13 is enriched in the *Drosophila* central nervous system. In homozygous *Vps13* mutant flies full length Vps13 protein levels were below the detection limit, visualized using Western blot analysis using the antibody against the C-terminal domain (Fig 1). The antibody directed against the N-terminal part of the protein, recognized a truncated Vps13 product in extracts of homozygous mutants, consistent with the presence of the Piggybac element insertion, indicating that the antibodies are specific, that the expression of full length Vps13 is strongly decreased and a truncated Vps13 product is present in mutant flies (Fig 1). Exact excision of the PiggyBac element in 3 independent lines resulted in recovery of the expression of a full length Vps13 protein in fly heads (Fig 1, S2 Fig). The excision lines were used as controls in further studies. These results indicate that the *Vps13* mutant line is a suitable tool to study the function of Vps13 in *Drosophila*.

### Vps13 co-fractionates with Rab7 and Rab5

**Fig 2:**
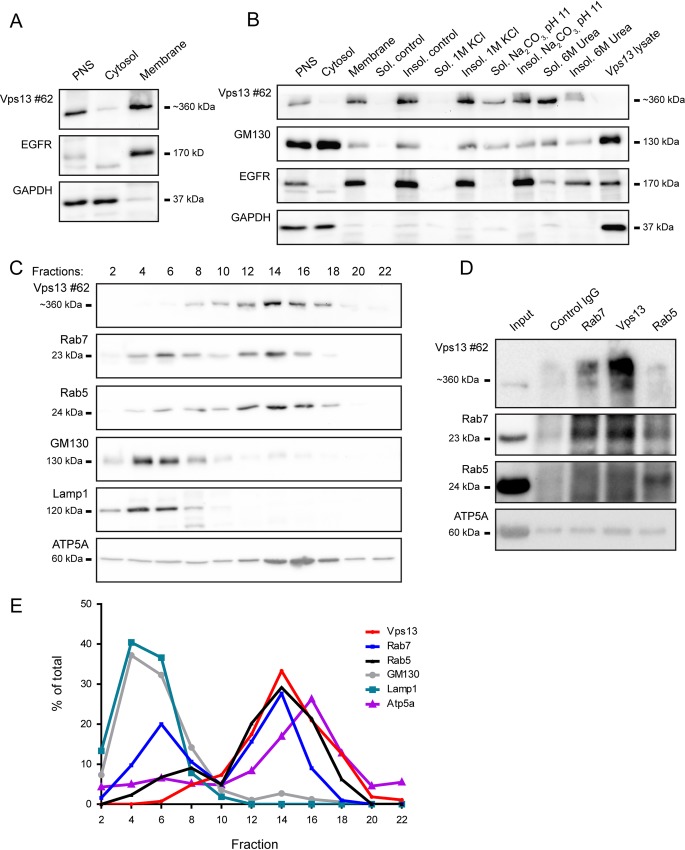
Vps13 co-fractionates with Rab7 and Rab5. (A) Western blot analysis of control fly head samples fractionated into a cytosolic and membrane fraction from postnuclear supernatant (PNS). EGFR was used as a membrane marker and GAPDH as a cytosolic marker. (B) Membrane fractions from control fly heads treated with 1 M KCl, Na_2_CO_3_ pH 11 or 6 M urea were centrifuged to separate the soluble and insoluble (membrane containing) fractions. The level of Vps13 was determined in these fractions. Markers for peripheral membrane proteins (GM130), integral membrane proteins (EGFR) and the cytosolic proteins (GAPDH) were used. The “*Vps13* lysate” lane contains a lysate derived from *Vps13* homozygous mutant fly heads, as expected no Vps13 is detected, demonstrating the specificity of the antibody against Vps13. (C) Membranes from control fly heads were fractionated on a sucrose gradient. Western blot analysis was performed to analyze the distribution of Vps13 in relation to markers associated with membranes of various organelles: Rab7 (late endosomes), Rab5 (early endosomes), GM130 (golgi), Lamp1 (lysosomes) and ATP5A (mitochondria). (D) Immunoisolation of membranes from fraction 14 of the sucrose gradient using Vps13 NT, Rab7 and Rab5 antibodies. (E) Quantification of the sucrose gradient fractionation of Fig 2C.

We aimed to determine the subcellular localization of Vps13 in brain tissue, however the antibodies which were generated against Vps13 failed to show a specific staining using immunolabeling. We therefore followed a cell fractionation approach to determine the subcellular localization of Vps13. We found that Vps13 was mainly, but not exclusively, present in the isolated membrane fraction (Fig 2). To determine whether Vps13 is a peripheral or integral membrane protein the membranes were treated with a variety of buffers to extract proteins as previously described [26]. High salt buffer could not remove Vps13 from the membrane fraction, while high pH and high concentration of urea did (Fig 2). This shows that Vps13 has characteristics similar to a peripheral membrane protein, such as Golgi Matrix protein 130 kDa (GM130) [26], but different from an integral membrane protein like Epidermal Growth Factor Receptor (EGFR), both of them were used as controls in these experiments (Fig 2). The membrane fraction was separated on a sucrose gradient and the distribution of Vps13 was determined in relation to marker proteins for various organelles. The distribution of Vps13 was different compared to the distribution of markers for Golgi (GM130), lysosomes (Lamp1) and mitochondria (ATP5A) (Fig 2). Vps13 was mainly present in fractions 12 to 16 in which also Rab5 and Rab7, Rab-GTPases involved in the regulation of endosomal trafficking, were present. Rab5 is mainly present on early endosomes and Rab7 is enriched on late endosomes [27]. To study this further, Rab7 positive membranes from fraction 14 were immuno-isolated and Vps13 was shown to be present in these samples (Fig 2). Furthermore, Rab7, but not Rab5 was enriched in membranes immuno-isolated with Vps13 antibodies (Fig 2). Together, these data suggest that Vps13 is a peripheral membrane protein associated with Rab7 positive membranes.

### *Vps13* mutant flies have a decreased life span and show age dependent neurodegeneration

**Fig 3:**
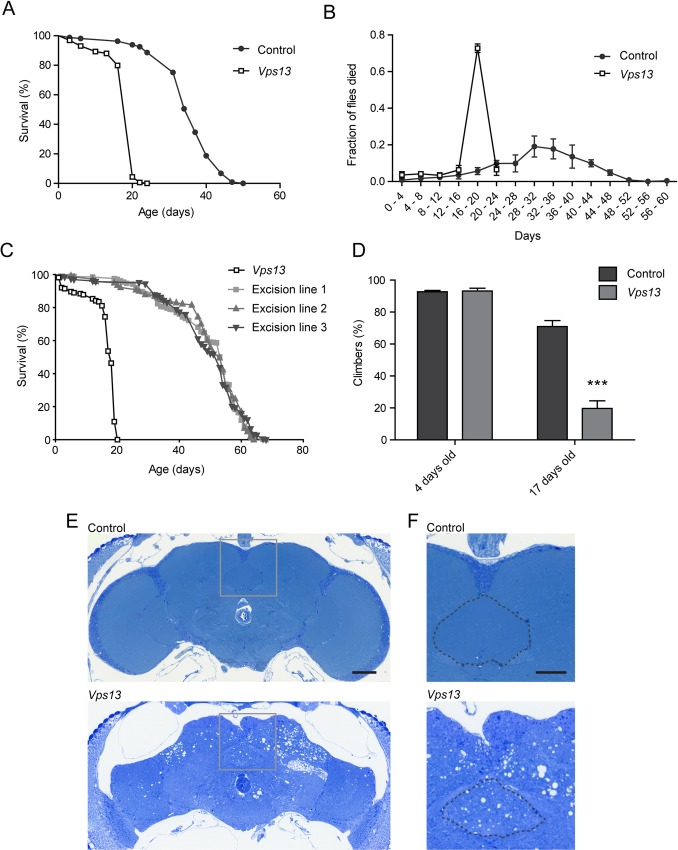
*Vps13* mutant flies show a decreased life span, age dependent impairment of locomotor function and neurodegeneration. (A) Life span analysis of isogenic control and *Vps13* mutant flies. (B) The fraction of dead flies of total flies used,observed within the indicated time intervals. (C) Life span curve of *Vps13* mutant flies and three excision lines. (D) Climbing behavior was analyzed by determining the percentage of isogenic control and *Vps13* mutant flies (4 and 17 days old) able to climb 5 cm against gravity within 15 seconds. Mean and SEM are plotted (n = 5). For statistical analysis a two-tailed students T-test was used. P<0.001 is ***. (E) Fly heads (20 day old) of control and homozygous *Vps13* mutant flies were fixed, dehydrated and embedded in epon. Sections, visualizing a cross section of the complete brain, were stained with toluidine blue. The scale bar indicates 50 μm.(F) Higher magnification images of the boxed area’s in Fig E. The central complex is denoted with a dotted line. The scale bar indicates 25 μm.

After validation of the *Drosophila Vps13* mutant and characterizing its subcellular localization, we investigated the physiological consequences of impaired Vsp13 function. Characteristics of several *Drosophila* models for neurodegenerative diseases are a decreased life span, impaired locomotor function and the presence of brain vacuoles [28]. As a control an isogenic fly line (*w*^*1118*^) and 3 independent precise excision lines were used. Homozygous *Vps13* mutant flies showed a decreased life span compared to isogenic controls and the excision lines (Fig 3, S1 Table). 75% of the mutant flies died between 16 and 20 days of age while control flies showed a more gradual decline (Fig 3). Young *Vps13* mutant flies showed climbing capabilities comparable to controls, however around day 17 the climbing ability of *Vps13* mutant flies was decreased (Fig 3, S1 Movie).

To further investigate neurodegenerative features in *Vps13* mutants, brain sections were analyzed by light microscopy and an increase in vacuoles was observed in brains of 20 day old flies while they were absent in brains from isogenic controls (Fig 3). Vacuoles in *Vps13* mutant flies were (among other regions) present in the central complex, known for its function in locomotor control (Fig 3) [29]. The impaired locomotor function upon ageing, shortened life span and the presence of large vacuoles in the brain of *Vps13* mutant flies are all characteristics of neurodegeneration in *Drosophila* [28].

### *Vps13* mutant flies show impaired protein homeostasis

**Fig 4:**
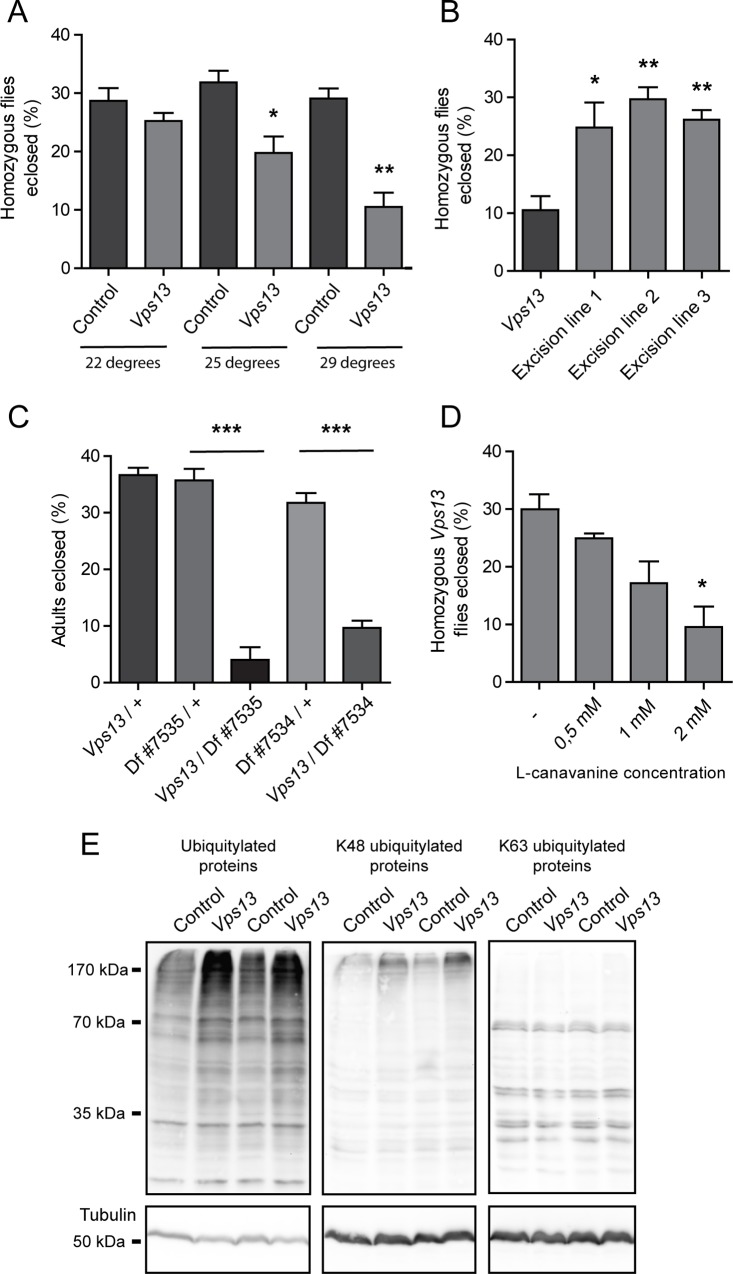
Impaired Vps13 function leads to defects in protein homeostasis. (A) Percentage of isogenic control and *Vps13* mutant flies that eclosed at increasing temperatures. (B) Percentage of homozygous *Vps13* mutant flies and excision line flies that eclosed at 29°C. (C) Percentage of flies of various genotypes that eclosed at 29°C. Two independent deficiency lines (lacking a genomic area containing the *Vps13* gene) were crossed with *Vps13/ CyO* heterozygous flies. Eclosion rate of the following genotypes was analyzed: *Vps13/+*, *Df #7535/+*, *Vps13/Df #7535*, *Df #7534/+* and *Vps13/Df #7534*. (D) Percentage of *Vps13* flies that eclosed at 22°C on food with increasing concentrations of L-canavanine. (E) Western blot analysis of lysates of 1 day old control and *Vps13* mutant fly heads. Ubiquitylated proteins, K48 ubiquitylated proteins and K63 ubiquitylated proteins were detected. All quantifications show the mean and SEM of at least three independent experiments per condition. For statistical analysis a two-tailed students T-test was used in combination with a Welch’s correction if necessary. P<0.05 is *, P<0.01 is ** and P<0.001 is ***.

Since neurodegenerative phenotypes are often linked to impaired protein homeostasis, we investigated the viability of *Vps13* mutants under proteotoxic stress by using a previously established eclosion assay [30]. The percentage of homozygous survivors was 19.76% of the total amount of eclosing flies at 25°C (Fig 4), which is less than the expected 33.3% according to Mendelian inheritance. This indicates that the viability of *Vps13* mutants was decreased compared to controls. To induce proteotoxic stress we analyzed the eclosion rate at increased temperature. The eclosion rate further decreased in a temperature dependent manner, indicating a temperature sensitivity of *Vps13* homozygous animals during development (Fig 4). As controls, the excision lines were tested and no decreased viability at 29°C was observed (Fig 4). Subsequent crosses with the *Vps13* allele over two deficiency lines lacking a genomic region including the *Vps13* gene (S2 Fig) also showed a decreased eclosion rate at 29°C (Fig 4), supporting the fact that temperature sensitivity is due to loss of Vps13 function.

To further investigate increased sensitivity to proteotoxic stress, *Vps13* mutant flies were fed with L-canavanine, an arginine analogue that induces protein misfolding, during development [31]. *Vps13* homozygous mutants showed an L-canavanine induced decrease in eclosion rate in a concentration dependent manner (Fig 4) indicating a defect in the ability of these flies to maintain protein homeostasis. Defects in cellular protein homeostasis are often associated with an accumulation of ubiquitylated proteins [32]. Indeed, extracts derived from *Vps13* mutant fly heads contained increased levels of ubiquitylated proteins compared to isogenic controls and excision lines (Fig 4, S2 Fig). Extracts derived from flies containing the *Vps13* allele over a deficiency chromosome gave comparable results (S2 Fig). Further specification revealed an increase in lysine K48 ubiquitylated high molecular weight (around 170 kDa) proteins, however no difference was observed in K63 ubiquitylated proteins (Fig 4). Because K48 ubiquitylated proteins are targeted for degradation, this accumulation may indicate that *Vps13* mutant flies suffer from an impairment in protein homeostasis [32].

### Protein aggregation in *Drosophila Vps13* mutant central nervous system

**Fig 5:**
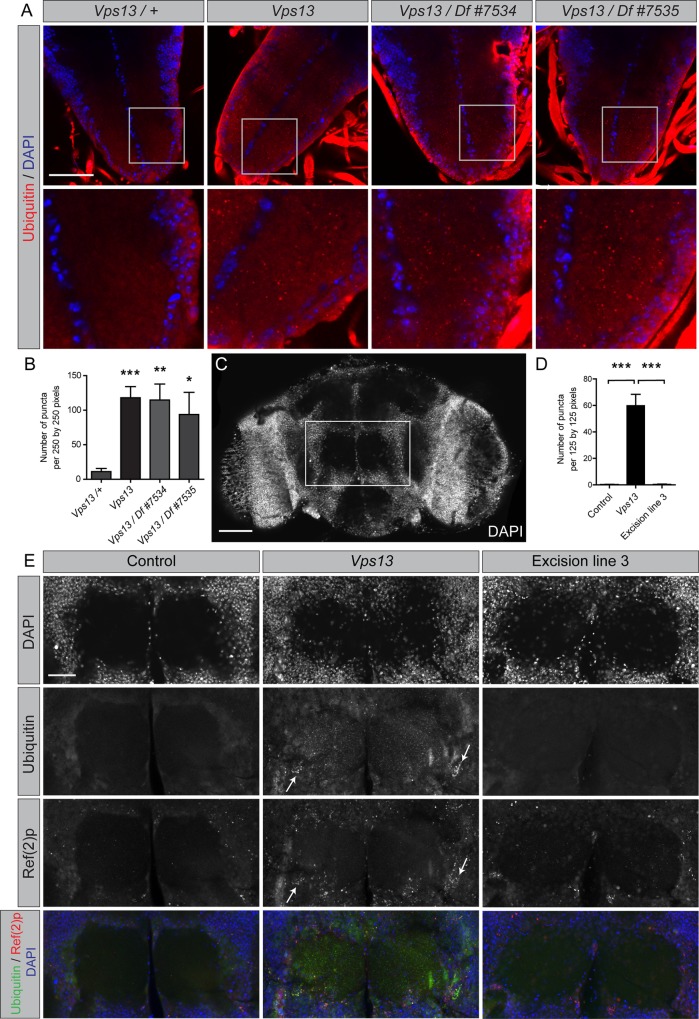
Central nervous system of larval and adult *Vps13* mutants contain protein aggregates. (A) Ventral nerve cords of control, *Vps13* mutant, *Vps13/Df #7534* and *Vps13/Df #7535* third instar larvae were stained for ubiquitylated proteins and DAPI. The presence of DAPI indicates areas where nuclei of neuronal cell bodies or glial cells are located. DAPI negative regions represent areas mainly containing axonal and synaptic structures [33,35]. The areas in the grey boxes are shown below as higher magnification images. The scale bar indicates 50 μm. (B) Quantification of the number of ubiquitylated protein puncta in the ventral nerve cord. (C) Staining of 1 day old adult control brains using DAPI. The grey box denotes the area in the brain where the two antennal lobes are located. The presence of DAPI indicates areas where nuclei of neuronal or glial cell bodies are located. The center area which is negative for DAPI contains axons and synaptic structures [34]. The scale bar indicates 50 μm. (D) Quantification of the number of puncta of ubiquitylated proteins in the antennal lobes derived from 1 day old isogenic controls, *Vps13* mutants and excision line 3. (E) Staining of brains derived from 1 day old controls, *Vps13* mutants and excision line 3 flies for ubiquitylated proteins, Ref(2)p and DAPI. The scale bar indicates 20 μm Arrows indicate colocalization of Ref(2)P and Ubiquitin positive foci. All quantifications show the mean and SEM of at least three independent experiments per condition. For statistical analysis a two-tailed students T-test was used in combination with a Welch’s correction if necessary. P<0.05 is *, P<0.01 is ** and P<0.001 is ***.

Impaired protein homeostasis often leads to the aggregation of proteins, therefore protein aggregation was investigated in *Vps13* mutants. Larval ventral nerve cords and brains from adult flies were dissected, fixed and stained for DAPI to visualize structures containing neuronal cell bodies (DAPI positive) and to visualize neuropils (DAPI negative), containing axons and dendrites [33–35]. The tissues were co-stained for Ubiquitin. Mainly neuropils in both larval ventral nerve cords and adult brains of *Vps13* mutants showed an increased number of ubiquitylated protein puncta compared to control (Fig 5 and S3 and S4 Figs). Furthermore, samples from *Vps13* mutant fly heads contained more Triton x-100 insoluble ubiquitylated proteins compared to controls (S3 Fig), indicating an accumulation of protein aggregates in *Vps13* mutants. Protein aggregation is often accompanied by an accumulation of Ref(2)P, the *Drosophila* orthologue of p62 [36]. Western blot analysis showed an increase in Ref(2)P in *Vps13* mutant fly heads compared to isogenic control and excision line fly heads (S2 Fig). Extracts derived from fly heads of the *Vps13* allele over a deficiency gave comparable results (S2 Fig). In addition a partial colocalization was observed between Ref(2)P and ubiquitin positive protein aggregates in *Vps13* mutant brains mainly in areas containing neuronal cell bodies (Fig 5, S4 Fig). This is consistent with published data showing the accumulation of ref(2)P in autophagy mutants and proteostasis mutants mainly in DAPI-positive areas [36]. These experiments show that *Vps13* mutant flies accumulate protein aggregates in the central nervous system of larvae and adult flies.

### *Vps13* mutant phenotypes are rescued by overexpression of HsVPS13A

**Fig 6:**
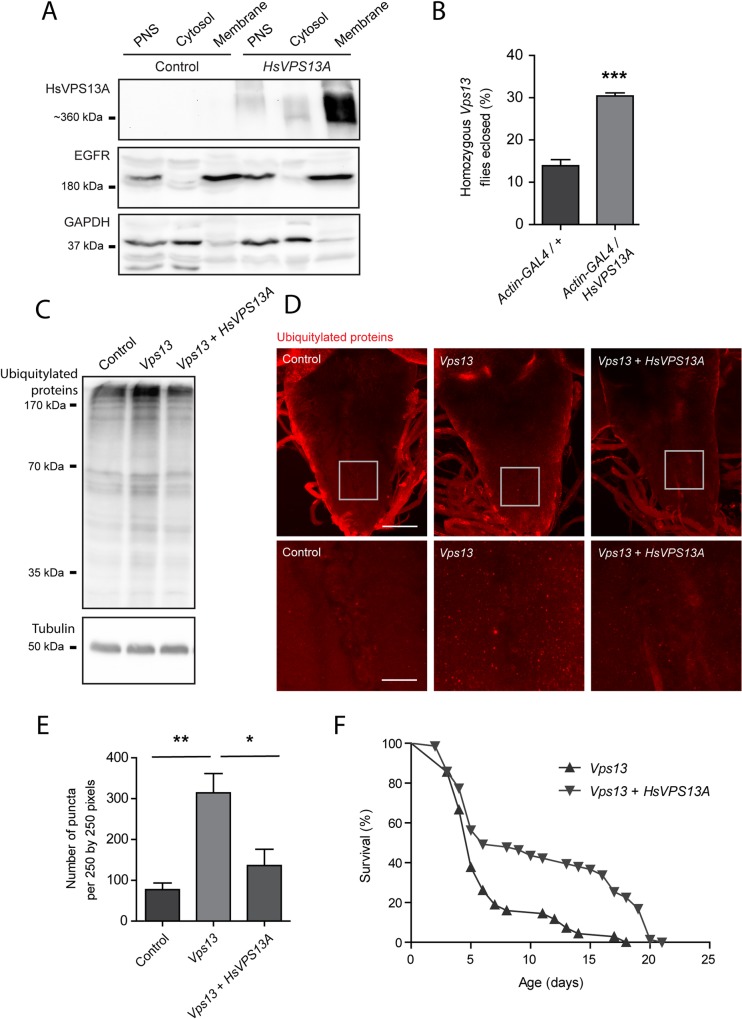
Overexpression of HsVps13A rescues phenotypes of *Vps13* mutants. (A) Samples from fly heads of *Actin-GAL4 / +* (as a control) and *Actin-GAL4 / UAS-HsVps13A* (HsVps13A expressing) flies were separated into a membrane and cytosol fraction and analyzed by Western blot for HsVps13A levels. EGFR and GAPDH used as controls for membrane and cytosolic proteins, respectively. (B) Eclosion rate of *Vps13* mutant flies a *Actin-GAL4/+* (control) or *Actin-GAL4/UAS-HsVp13A (HsVps13A expressing)* background at 25°C. (C) Ubiquitylated proteins from samples of 1 day old fly head extracts *of Vps13/CyO; Actin-GAL4/+* (as a control), *Vps13/ Vps13; Actin-GAL4/+* (representing homozygous mutants) and *Vps13/ Vps13; Actin-GAL4/UAS-HsVps13A* (representing homozygous mutants expressing human VPS13A). (D) Representative picture of ubiquitylated protein staining of the third instar larval ventral nerve cord of *Vps13/CyO; Actin-GAL4/+* (as a control), *Vps13/ Vps13; Actin-GAL4/+* and *Vps13/ Vps13; Actin-GAL4/UAS-HsVps13A*. Arrows indicate accumulations of ubiquitylated positive structures. The scale bar indicates 50 μm and 12,5 μm in the enlargement. (E) Quantification of the number of puncta in third instar larval ventral nerve cord of the experiment presented in Fig 6D. (F) Life span curve of *Vps13/ Vps13; Actin-GAL4/+* and *Vps13/ Vps13; Actin-GAL4/UAS-HsVps13A*. All quantifications show the mean and SEM of at least three independent experiments per condition. For statistical analysis a two-tailed students T-test was used in combination with a Welch’s correction if necessary. P<0.05 is *, P<0.01 is ** and P<0.001 is ***.

Homozygous *Vps13* mutants display various characteristics indicative of neurodegeneration accompanied by an impairment in protein homeostasis. To further validate our model and investigate its relevance for HsVPS13A function we overexpressed HsVPS13A in the *Vps13* mutant background. The sequences of Vps13 and HsVPS13A show 29% identity, while the N-terminal chorein domains have an identity of 50% (S1 Fig). Using the UAS-GAL4 system [37] HsVPS13A was ubiquitously overexpressed in the *Drosophila Vps13* mutant background to investigate whether this could rescue the phenotypes observed in the *Vps13* mutant flies. Fractionation and Western blot analysis, using an antibody against the HsVPS13A protein, showed that HsVPS13A was expressed in the transgenic flies and was mainly present in the membrane fraction (Fig 6). Overexpression of HsVPS13A in the *Vps13* mutant background increased the viability (Fig 6), reduced the amount of ubiquitylated proteins (Fig 6) and decreased the number of ubiquitylated protein puncta in the larval ventral nerve cord (Fig 6). In addition, overexpression of HsVPS13A extended the life span of *Vps13* mutant flies (Fig 6, S1 Table). These results indicate not only a structural conservation but also a functional conservation between the human VPS13A and the *Drosophila* Vps13 protein for at least a subset of the functions of these proteins.

## Discussion

ChAc is a recessively inherited neurodegenerative disorder caused by loss of function mutations in the *HsVPS13A* gene [3,4]. The study of HsVPS13A function and the pathological mechanisms playing a role in ChAc is hampered by the limited availability of multicellular models for ChAc. Although Vps13A knock-out ChAc mouse models were generated, they possess variable or no abnormalities in brain tissue depending on the genetic background [24]. This underscores the complexity of studying VPS13A in the central nervous system and suggests the presence of genetic factors playing a role in the phenotype induced by impaired function of VPS13A in the brain. The goal of this study was to use *Drosophila melanogaster* to establish a relatively simple multicellular model for ChAc and study the consequences of Vps13 dysfunction in the ageing central nervous system.

We established a *Drosophila melanogaster* model for ChAc by using *Vps13* mutant flies which express a truncated Vps13 protein. ChAc has been shown to be caused by loss-of-function mutations, most of them leading to total absence of protein [5]. In addition, alteration of the most C-terminal region of the main protein isoform, leading to the presence of a truncated protein, have also been found ([5], Velayos-Baeza et al, unpublished results). Although a detailed phenotypic study of ChAc patients comparing consequences of no protein or a truncated protein present, has not been performed, it can be concluded that the main features of the disease are present in all cases regardless the presence of a truncated protein or the absence of VPS13A protein. The presented *Drosophila* model may be of use for future studies to investigate effects of various specific mutations in the *VPS13A* gene and how this affects protein homeostasis, neurodegeneration and life span. The *Vps13* mutant flies show progressive neurodegenerative phenotypes such as a shortened life span, impaired locomotor function and the presence of vacuoles in brain tissue at older age [28]. These phenotypes are accompanied by defects in protein homeostasis and by accumulation of protein aggregates in the central nervous system. Many neurodegenerative diseases are characterized by defects in protein homeostasis and the accumulation of protein aggregates in the brain [38]. It will be of interest to investigate protein homeostasis and the presence of p62 positive protein aggregates in ChAc mouse models or in post-mortem tissue of affected ChAc individuals.

Our results demonstrate that *Drosophila* Vps13 is a peripheral membrane protein associated with Rab7 positive membranes. Rab7 positive late endosomes are involved in lysosomal protein degradation pathways such as autophagy and phagocytosis [39], suggesting a role of *Drosophila* Vps13 in the lysosomal degradation pathway. This is consistent with findings that knock down of HsVPS13A is associated with impaired autophagic degradation in HeLa cells [8]. In addition, an accumulation of Ref(2)P and colocalization with Ubiquitin positive dots was also observed in *Drosophila* autophagy mutants [36], further suggesting a role for Vps13 in autophagy. It should be stressed however that Ref(2)P accumulation and colocalization with Ubiquitin also occurs when proteosomal degradation is impaired. Future research is therefore required to determine whether impaired autophagy or impaired proteosomal degradation (or both) lay at the base for the disturbed protein homeostasis in *Vps13* mutants. Furthermore, a study in *Tetrahymena thermophila* suggests a potential role for VPS13 in phagocytosis [7]. Together, these studies show that a number of lysosomal degradation pathways may potentially be affected by VPS13 dysfunction, and together this could contribute to the impaired protein homeostasis in *Vps13* mutants. In an ageing organism damaged proteins may accumulate and cause the observed neurodegenerative phenotype [40]. Future research is required to show whether *Drosophila* and human VPS13A play a role in a single or in multiple lysosomal degradation pathways and whether in the nervous system VPS13 is linked to membrane contact sites, as was demonstrated for VPS13 in yeast [20,21].

The *Drosophila Vps13* mutant phenotype was partly rescued by human VPS13A, demonstrating at least a degree of functional conservation between flies and human. The availability of our characterized *Drosophila* model will enable future genetic screens to find modifiers of ChAc and will enable screens for chemical compounds that rescue one or multiple pertinent phenotype(s) of neurodegeneration.

## Materials and Methods

### Fly stocks and genetics

Fly stocks were maintained and experiments were done at 25°C on standard agar food unless otherwise indicated. The *Vps13{PB}c03628* stock was acquired from the Exelixis stock centre [25] and isogenized to the *w*^*1118*^ stock. The generation of the isogenic controls was performed as previously described [41]. In short, The isogenic fly lines that serve as a control were generated by backcrossing the *Vps13* mutant line for 6 generations with the control stock (*w*^*1118*^). Backcrossing the mutant line for 6 generations is required to remove background mutations and isogenized control stocks are being generated and used as controls in all experiments. The following stocks were acquired from the Bloomington Stock Centre: *w^1118^*; *CyO*, *P{Tub-PBac\T}2/wgSp-1* (8285), *Df(2R)Exel6053* (7535), *Df(2R)Exel6052* (7534), *Actin-GAL4/Tm6B* (3954).

The *Vps13{PB}c03628* excision lines were created by crossing the *Vps13{PB}c03628* stock with the PiggyBac transposase overexpressing fly line (Bloomington stockcenter; #8285) to remove the Piggybac insertion. The acquired “Hopout” chromosomes of these excision lines were balanced over *CyO* and three independent offspring lines balanced over *CyO* were established. They are referred to as excision lines 1 to 3.

### Generation of HsVPS13A expression flies

The full-length cDNA of the human *VPS13A* gene, variant 1A, corresponding to positions 252 to 9907 of GeneBank NM_033305 (but containing synonymous SNPs rs17423984 (A5583G, Thr1861Thr) and rs3737289 (A9069G, Gly3023Gly), was available after combination of several fragments amplified by RT-PCR [6] and cloning into pcDNA4-TO-mycHis (Invitrogen). To obtain a plasmid for expression of *HsVPS13A* in *D*. *melanogaster*, the above insert was transferred to vector pUAST [37]. The plasmid was sent to Bestgene for embryo injection and generation of the transgenic flies.

### Physiological assays

Crosses for the life span assays were performed at 25°C and offspring were selected 24 hours after the start of eclosion. 10 to 20 flies per tube were housed at 25°C and put into fresh vials every 3 or 4 days. The incidence of dead flies was counted at least every 4 days. Life spans were repeated at least three times.

The climbing assay was performed with at least 5 vials with 10 to 15 flies each. The flies were tapped to the bottom and the amount of flies that reached 5 cm within 15 seconds was noted as climbers and flies under the 5 cm mark were scored as non-climbers. Experiments were repeated three times.

Crosses to determine the eclosion rate were performed with 10 female and 5 male flies. The flies were allowed to mate for 48 hours on Bloomington food at the indicated temperatures. The amount of offspring of the indicated genotypes was determined 5 days after eclosion of the first progeny. L-Canavanine (Sigma), was mixed with the food at the indicated final concentrations. Sensitivity to L-Canavanine was determined as previously described [30]. In short: heterozygous *Vps13* males and females (flies carrying a chromosome containing the *Vps13* mutation over a balancer chromosome (*Vps13*/CyO)) were allowed to mate and the number of homozygous *Vps13* mutant progeny was determined and given as percentage of the total progeny (sum of heterozygous (*Vps13*/Cyo) plus homozygous progeny (*Vps13/Vps13*)). Under control conditions the percentage homozygous *Vps13* eclosing progeny is 33% (because the CyO/CyO genotype causes lethality). To determine the eclosion rate of combinations of different alleles, the alleles under investigation (*Vps13*, *w*^*1118*^ or one of the deficiency lines) were balanced over *CyO* and mated with each other (e.g. *Vps13/CyO* x *Df #7535/CyO*). Based on Mendelian laws, the percentage of non-CyO progeny flies from these crosses is around 33%. When the viability of the non-*CyO* flies is compromised, the percentage non-CyO eclosing flies is lower than the expected 33% of the total eclosing flies. For all eclosion experiments more than 100 eclosed flies were scored per condition. Due to toxicity of the *Actin-GAL4* driver in the *Vps13* background, all rescue experiments using human VPS13A were performed at 25°C.

### Western blot analysis

Flies were flash frozen in liquid nitrogen and heads were separated from bodies by using a vortexer. 30 μl freshly prepared Laemmli buffer (2% SDS, 5% 2-mercaptoethanol, 10% glycerol, 0.004% bromophenol blue, 0.0625 M Tris HCl pH 6.8) was added per 10 heads and the samples were sonicated three times for 5 seconds on ice. 5% 2-mercapthoethanol (Sigma) was added and the samples were subsequently boiled for 5 minutes. Samples were run on 12% polyacrylamide gels and transferred onto nitrocellulose membranes. For Vps13 detection the samples were prepared using 2x Laemmli buffer without 2-mercaptoethanol containing 0,8 M urea and 50 mM DTT. The samples were run on a 6% polyacrylamide gel and blotted overnight using transfer buffer containing 10% methanol. Membranes were incubated in 5% milk in PBS 0,1% Tween-20 and subsequently stained using the primary antibody in PBS 0.1% Tween-20 over night at 4°C. Staining with secondary antibodies (1:4000, GE Healthcare) was done at room temperature in PBS 0.1% Tween-20. Signal on membranes was visualized using ECL or super-ECL solution (Thermo Scientific) in the dark room, the GeneGnome (Westburg) or the ChemiDoc Touch (BioRad).

The following antibodies were used for Western blot analysis: beta-actin (1:2000, Cell Signalling, #3700), alpha-tubulin (1:4000, Sigma, T5168), ubiquitylated proteins (1:1000, FK2, Enzo life sciences, BML-PW8810-0500), K48-ubiquitinated proteins (1:1000, Cell signalling, #8081), K68-ubiquitinated proteins (1:1000, Cell signaling, #5621), Ref(2)p [42], HsVPS13A (1:1000, Sigma, HPA021662), EGFR (1:1000, Santa Cruz Biotechnology, sc-03-G), GAPDH (1:1000, Novus biologicals, NB100-56875), GM130 (1:2000, Abcam, ab30637), Rab5 (1:1000, Abcam, ab31261), Rab7 (1:1000, [43], ATP5A (1:5000, Mitoscience via Abcam, MS507) and Lamp1 (1:1000, Abcam, ab25630).

### Generation of *Drosophila* Vps13 antibodies

The Vps13 #62 antibody was made by immunizing rabbits with a synthetic peptide containing the amino acids 3299 to 3314 of Vps13 (Eurogentec). A dilution of 1:1000 was used for Western blot experiments.

For the Vps13 NT antibody *Vps13* cDNA corresponding to amino acids 576–976 was cloned in pET28a (Novagen) to generate a His-Tag fusion protein that was expressed and purified with Ni-NTA resin (Qiagen) following manufacturer's instructions. The resulting recombinant protein was used to immunize rabbits. This antibody was used in a 1:1000 dilution for Western blot analysis.

### TX-100 detergent fractionation

Separation of the Triton X-100 insoluble and soluble fractions of fly heads was performed as described in [44]. In short, fly heads of 7 day old flies were separated from the bodies by freezing in liquid nitrogen and subsequent vortexing. The heads were kept on ice and homogenized with a pellet pestle in 1% Triton X-100 in PBS containing protease inhibitors. The sample was centrifuged at 4°C at 20800 g for 10 minutes. The supernatant was removed and the samples were washed in 1% Triton X-100 in PBS containing protease inhibitors. After a second centrifugation step the supernatant was removed, 5% Laemmli buffer was added and the sample was sonicated on ice. 5% beta-mercapthoethanol was added and the sample was boiled for 10 minutes.

### Cytosol vs Membrane fractionation and membrane extraction

A slightly modified protocol [45] was used. Approximately 800 fly heads were resuspended in 800 μl homogenization buffer HB (50mM Tris HCl pH 7.5, 150 mM NaCl, 1 mM EDTA, Protease inhibitor) and mechanically shredded using a pellet pestle motor (Kontes). The nuclei and intact cells were pelleted by centrifugation 5 min at 800 g, and the resulting postnuclear supernatant (PNS) was applied to ultracentrifugation at 100,000 g for 1 h using a TLA 100.3 rotor to generate the cytosol (C) and the membrane fraction (M). To analyze the association of Vps13 with membranes, the membrane fraction was treated with HB, 1 M KCl, 0.2 M sodium carbonate (pH 11), or 6 M urea for 45 min on ice, and then separated into a supernatant (Soluble) or a pellet (Insoluble) fraction by centrifugation at 4°C, 100,000 g for 1 h. Laemmli sample buffer was added to the insoluble and soluble fractions and the samples were processed for Western blot analysis.

### Subcellular fractionation and immunoisolation

A protocol based on Silvis et al. [46] was used. In short: For subcellular fractionation around 1000 fly heads were resuspended in 1 ml of homogenization buffer HB (50mM Tris HCl pH 7,5, 150mM NaCl, 1mM EDTA, Protease inhibitor, 0.25 M sucrose). The fly heads were homogenized by 20 strokes of a Potter-Elvehjem PTFE pestle and centrifuged at 800 g for 5 min, the pellet was discarded and the supernatant (post nuclear supernatant, PNS) was collected. The fly heads PNS was then pipeted onto a sucrose gradient containing 5%, 17.5%, 30%, 42.5%, 55% (w/v) in HB, the volume was 2 ml per concentration, and the gradient was spun at 4°C at 274 000 g for 4 h using a swinging bucket SW41 rotor in a Sorvall Discovery 90se. Fractions of 0.5 ml were harvested top to bottom from the gradient and transferred into 1.5 ml microcentrifuge tubes. The proteins present in each fraction were precipitated and concentrated using TCA, resuspendend in 75 μl of sample buffer, processed for Western blot analysis as described before and analyzed by Western blot. All the procedures were performed on ice.

To perform the immunoisolation, a Vps13-enriched fraction containing vesicles positive for markers of the early and late endosomal populations was obtained as described above. The Vps13 enriched fraction (30% sucrose) was collected (approximately 1 ml). Rabbit anti-Rab7, anti-Rab5, anti Vps13 NT, or a nonspecific rabbit IgG was added to the Vps13 enriched fraction and incubated overnight at 4°C with rotation. In addition, 30 μl A/G plus agarose beads per condition were washed with 1% BSA/HB three times and incubated with 1 ml 1% BSA/HB overnight at 4°C. The following day the beads were recovered and resuspended in 30 μl of HB per condition. 30 μl of the blocked and washed beads were then added and incubated with each condition for 3 h at 4°C with rotation. The bead–antibody–organelle complexes were collected and washed five times with HB. Laemmli sample buffer was added to the immunoisolated complexes, and samples were analyzed using Western blot analysis to detect the indicatedproteins.

### Q-PCR

RNA was extracted from whole flies (RNeasy purification kit) and transcribed into cDNA (M-MLV, Invitrogen). Q-PCR was done using Sybergreen (Biorad) and a Biorad i-cycler. The primers were directed to a sequence downstream of the PiggyBac insertion. RP49 mRNA levels were used for normalization. The following primers were used for *Vps13* mRNA: For–AGACGTGCCTGGGTCTAT and Rev–AAGGCTCGTGAGAGGTAC; and for *RP49* mRNA: For–GCACCAAGCACTTCATCC and Rev–CGATCTCGCCGCAGTAAA.

### Immunofluorescence

Adult and L3 larval brains were dissected in PBS and directly put on ice. The brains were fixed for 20 minutes in 3.7% formaldehyde and subsequently washed 3 times 10 minutes in PBS 0.1% Triton X-100, followed by an optional 1 hour blocking at room temperature in 10% normal goat serum in PBS 0.1% Triton X-100. Primary antibodies: ubiquitylated proteins (1:200, FK2, Enzo life sciences, BML-PW8810-0500) and Ref(2)P (1:1000) [42]. A Leica SP8 CLSM, and a Zeiss-LSM780 NLO confocal microscope were used to obtain the fluorescent images.

### Histology

Flies were sedated using CO_2_, the proboscis was removed and the flies were decapitated. The heads were immediately transferred into fixative at 4°C containing 2% Glutaraldehyde, 0.2% picric acid and 4% paraformaldehyde in 0.1 M Cacodylate buffer. The fly heads were fixed at 4°C on a rotator for at least 48 hrs followed by three wash steps with Cacodylate buffer. Next, the heads were transferred to postfix (1% osmium tetroxide and 1,5% potassiumferrocyanide in 0.1 M Cacodylate buffer) for 2hrs at 4°C and washed with ddH_2_O, dehydrated using an ethanol series and embedded in Epon. Thick sections were produced using a Leica EM UC7 Ultramicrotome, sections were transferred to glass slides, stained using Toluidine blue and imaged with an Olympus BX50 light microscope.

### Quantifications and statistical analysis

Quantification of images obtained by immunohistochemistry (ubiquitylated proteins and Ref(2)p accumulations) of the central nervous system were blindly scored. In ImageJ a region of 250 by 250 pixels in the center of the brain was selected to exclude the background fluorescence at the edges of the brains. Subsequently the puncta were counted using the “find maxima” function.

The statistical significance of the data was calculated using the Student’s t-test (2-tailed and where appropriate with welches correction). Plotted values show the average of at least 3 independent experiments and error bars show the standard error of the mean. P-values below 0,05 were considered significant. In the figures P≤0,05 is indicated by a *, P≤0,01 by ** and P≤0,001 by ***.

Significance of the life span analyses was calculated with Graphpad prism5 using a Log-rank (Mantel-Cox) Test and a Gehan-Breslow-Wilcoxon Test. Graphs and life span curves were made using Graphpad prism5.

## Supporting Information

S1 Fig Alignment of Vps13 and HsVps13A. Identical amino acids are indicated in red. The conserved “Chorein domain” is indicated.
(TIF)

S2 Fig Western analysis of *Vps13*, excision lines and deficiency lines. (A) Western blot analysis of Vps13 protein level in isogenic control, *Vps13* mutant and excision line fly heads using the Vps13 #62 antibody. Tubulin was used as a loading control. (B) Western blot analysis of ubiquitylated proteins and Ref(2)p in control, *Vps13* mutant and excision line fly heads. Tubulin was used as a loading control. (C) Western blot analysis of Vps13 protein in *Vps13/+*, *Vps13*, *Vps13/Df #7534* and *Vps13/Df #7535* fly heads using the Vps13 #62 antibody. Tubulin was used as a loading control. (D) Western blot analysis of ubiquitylated proteins and Ref(2)p in *Vps13/+*, *Vps13*, *Vps13/Df #7534* and *Vps13/Df #7535* fly heads. Tubulin was used as a loading control.
(TIF)

S3 Fig Accumulation of puncta of ubiquitylated protein in the larval ventral nerve cord. (A) Ventral nerve cords of control and *Vps13* mutant third instar larvae were stained for ubiquitylated proteins. The areas in the grey boxes are shown below in higher magnification. Quantification of the number of puncta in the ventral nerve cord is given. The scale bar indicates 50 μm and 12.5 μm in the enlargement. (B) Quantification of the number of puncta present in a 250 by 250 pixel section of larval ventral nerve cord as depicted in 5A. (C) Triton x-100 fractionation of samples from control and Vps13 mutant fly heads analyzed for the levels of Triton x-100 insoluble ubiquitylated proteins. The quantification shows the mean and SEM of at least five larval ventral nerve cord stainings per condition. For statistical analysis a two-tailed students T-test was used in combination with a Welch’s correction if necessary. P<0.01 is **.
(TIF)

S4 Fig Accumulation of ubiquitylated protein puncta in the adult eye lobe. Stainings of the eye lobes of 1 day old adult control, *Vps13* mutant and excision line 3 stained for ubiquitylated proteins, Ref(2)p and DAPI. The higher magnification pictures are of the areas in the grey boxes. Arrows indicate colocalization of Ref(2)P and Ubiquitin positive foci. The scale bar in the overview picture indicates 50 μm and the scale bar in the zoom in indicates 20 μm.
(TIF)

S1 Table Details of the life span experiments presented. Depicted per figure are the fly lines used, the number of flies used for the experiment, the median life span, the Mantel-Cox test (M-C test) and Gehan-Breslow-Wilcoxon test (G-B-W test).
(TIF)

S1 Movie Movie of a climbing assay conducted with the control and *Vps13* mutant flies. (MP4)
