# Supplementary figures and images for: Drosophila Vps13 Is Required for Protein Homeostasis in the Brain
Source: PLoS One. 2017 Jan 20;12(1):e0170106. doi: 10.1371/journal.pone.0170106 (PMC5249141; doi:10.1371/journal.pone.0170106)

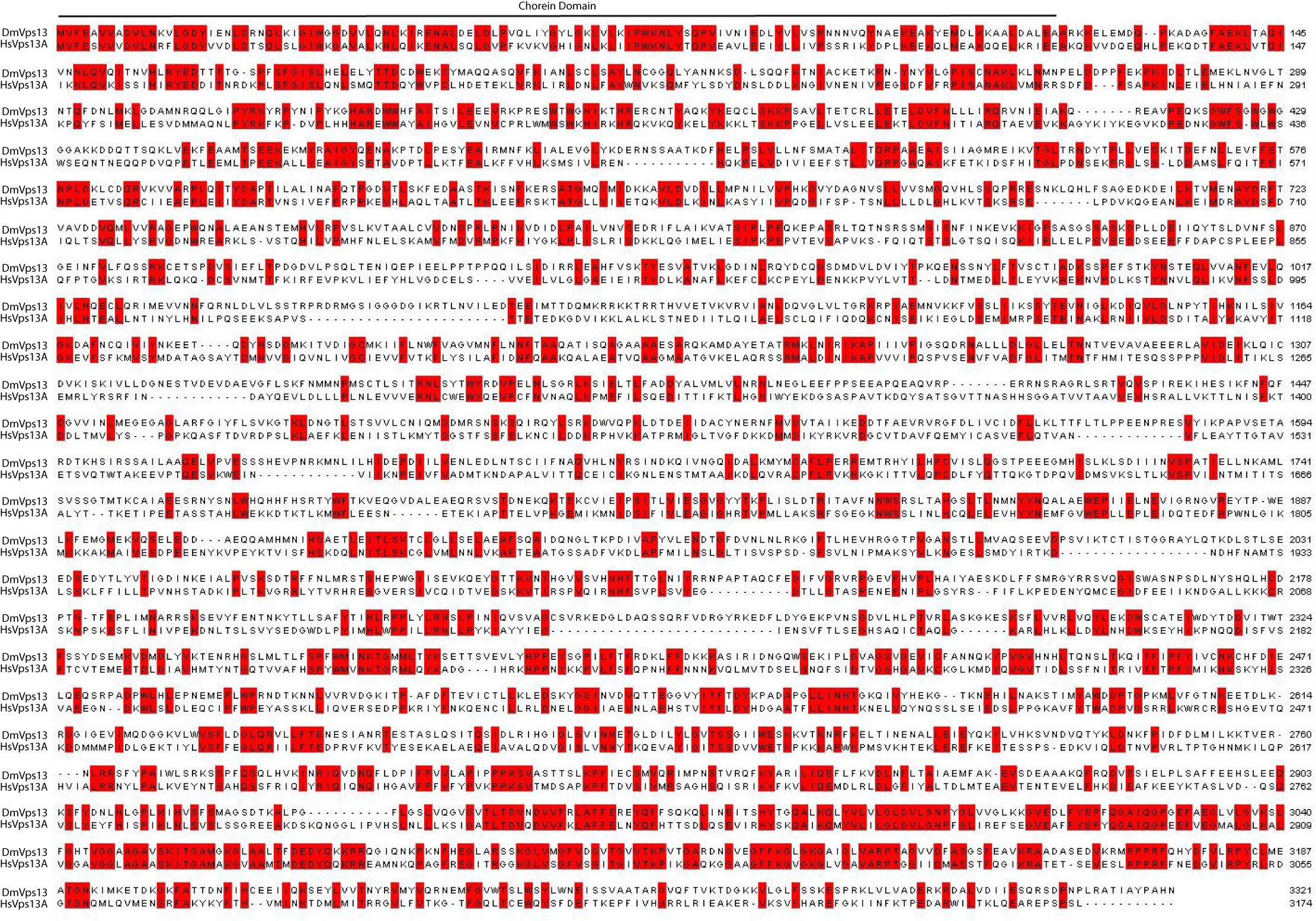

Supplement: S1 Fig — Identical amino acids are indicated in red. The conserved “Chorein domain” is indicated. (TIF) [file pone.0170106.s001.tif]

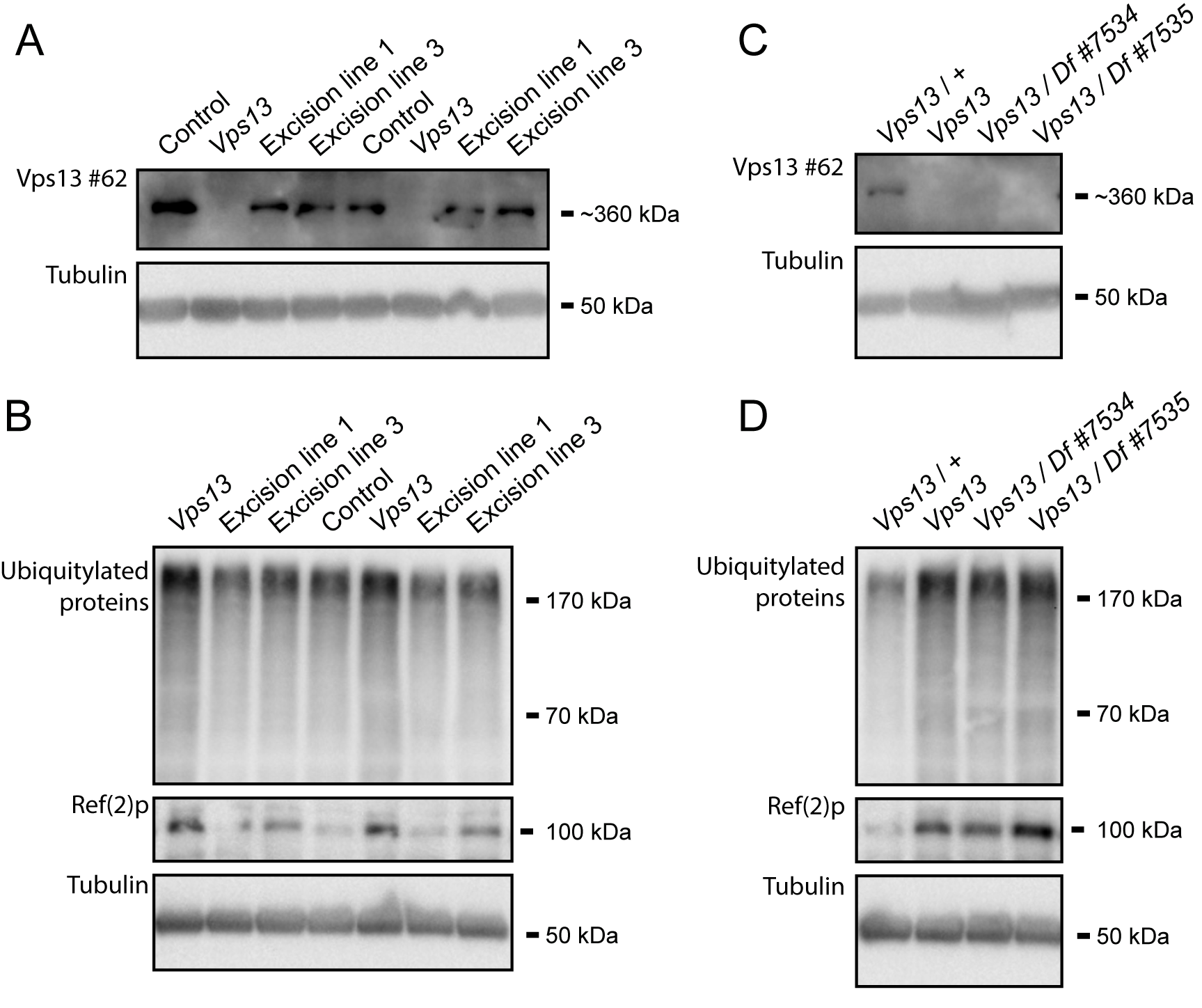

Supplement: S2 Fig — (A) Western blot analysis of Vps13 protein level in isogenic control, Vps13 mutant and excision line fly heads using the Vps13 #62 antibody. Tubulin was used as a loading control. (B) Western blot analysis of ubiquitylated proteins and Ref(2)p in control, Vps13 mutant and excision line fly heads. Tubulin was used as a loading control. (C) Western blot analysis of Vps13 protein in Vps13/+, Vps13, Vps13/Df #7534 and Vps13/Df #7535 fly heads using the Vps13 #62 antibody. Tubulin was used as a loading control. (D) Western blot analysis of ubiquitylated proteins and Ref(2)p in Vps13/+, Vps13, Vps13/Df #7534 and Vps13/Df #7535 fly heads. Tubulin was used as a loading control. (TIF) [file pone.0170106.s002.tif]

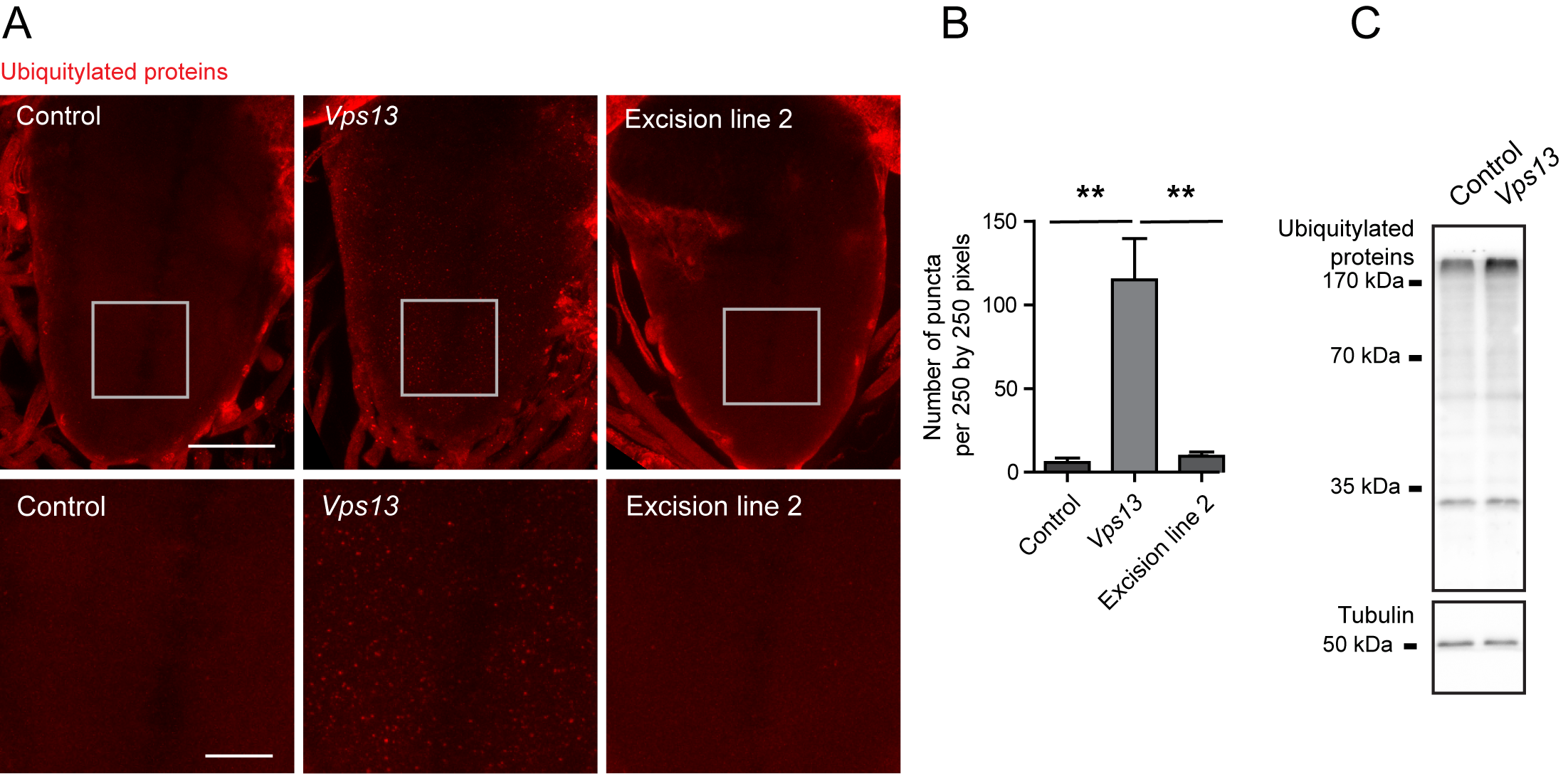

Supplement: S3 Fig — (A) Ventral nerve cords of control and Vps13 mutant third instar larvae were stained for ubiquitylated proteins. The areas in the grey boxes are shown below in higher magnification. Quantification of the number of puncta in the ventral nerve cord is given. The scale bar indicates 50 μm and 12.5 μm in the enlargement. (B) Quantification of the number of puncta present in a 250 by 250 pixel section of larval ventral nerve cord as depicted in 5A. (C) Triton x-100 fractionation of samples from control and Vps13 mutant fly heads analyzed for the levels of Triton x-100 insoluble ubiquitylated proteins. The quantification shows the mean and SEM of at least five larval ventral nerve cord stainings per condition. For statistical analysis a two-tailed students T-test was used in combination with a Welch’s correction if necessary. P<0.01 is **. (TIF) [file pone.0170106.s003.tif]

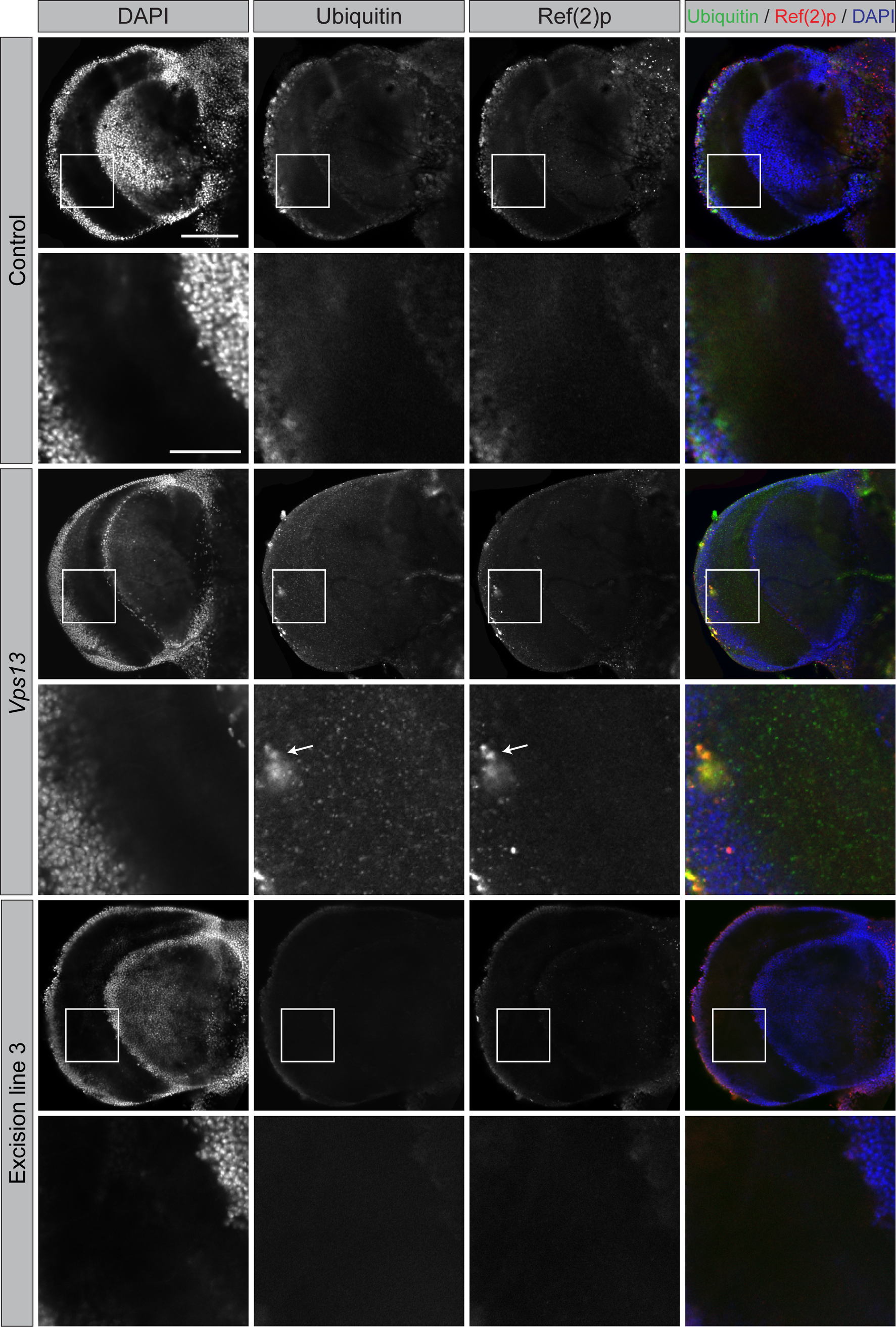

Supplement: S4 Fig — Stainings of the eye lobes of 1 day old adult control, Vps13 mutant and excision line 3 stained for ubiquitylated proteins, Ref(2)p and DAPI. The higher magnification pictures are of the areas in the grey boxes. Arrows indicate colocalization of Ref(2)P and Ubiquitin positive foci. The scale bar in the overview picture indicates 50 μm and the scale bar in the zoom in indicates 20 μm. (TIF) [file pone.0170106.s004.tif]

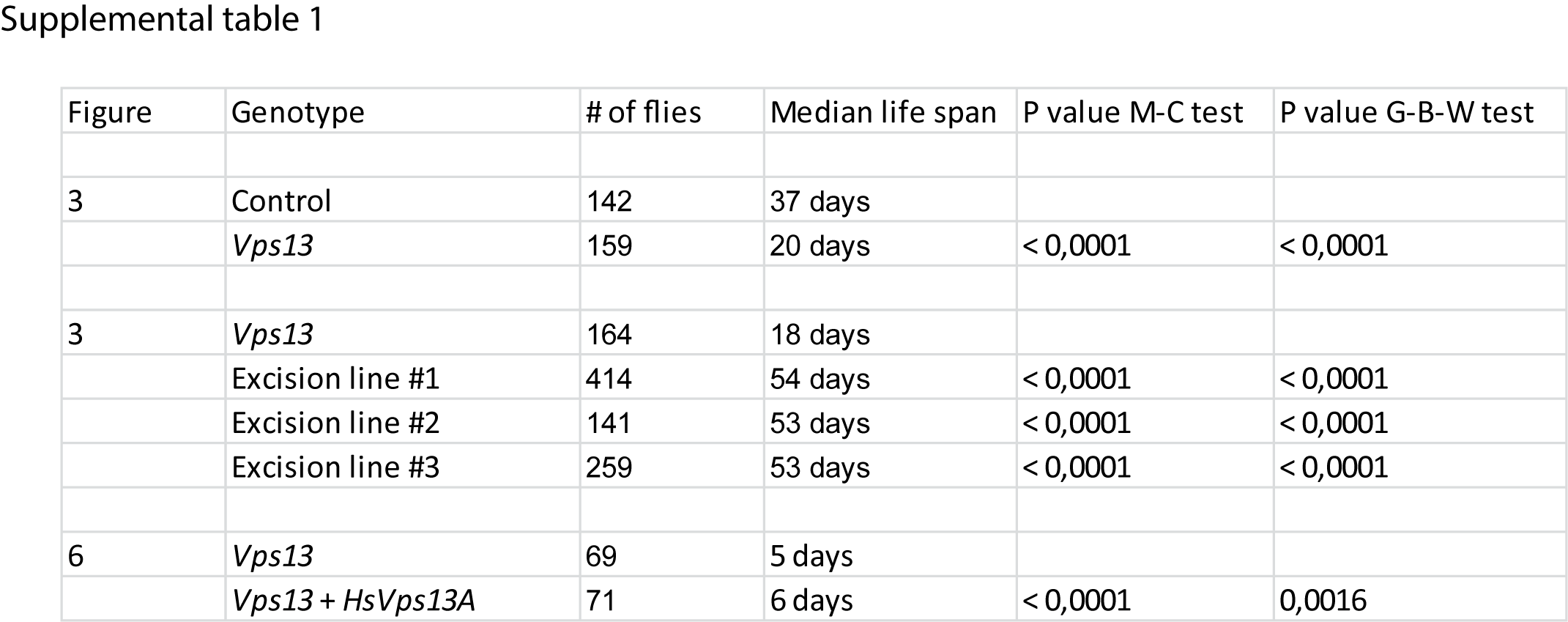

Supplement: S1 Table — Depicted per figure are the fly lines used, the number of flies used for the experiment, the median life span, the Mantel-Cox test (M-C test) and Gehan-Breslow-Wilcoxon test (G-B-W test). (TIF) [file pone.0170106.s005.tif]
